# An Improved *in Vivo* Deuterium Labeling Method for Measuring the Biosynthetic Rate of Cytokinins

**DOI:** 10.3390/molecules15129214

**Published:** 2010-12-15

**Authors:** Petr Tarkowski, Kristýna Floková, Kateřina Václavíková, Pavel Jaworek, Martin Raus, Anders Nordström, Ondřej Novák, Karel Doležal, Marek Šebela, Jitka Frébortová

**Affiliations:** 1 Centre of the Region Haná for Biotechnological and Agricultural Research, Faculty of Science, Palacký University, Šlechtitelů 11, 783 71 Olomouc, Czech Republic; E-Mails: karel.dolezal@upol.cz (K.D.); marek.sebela@upol.cz (M.Š.); jitka.frebortova@upol.cz (J.F.); 2 Department of Biochemistry, Faculty of Science, Palacký University, Šlechtitelů 11, 783 71 Olomouc, Czech Republic; E-Mails: DrobRangers@seznam.cz (K.F.); katka.vaclavik@seznam.cz (K.V.); p.jaworek@seznam.cz (P.J.); martin_raus@post.cz (M.R.); 3 Department for Oncology–Pathology, Karolinska Biomics Center, Karolinska Institutet, Z5:02, 17176 Stockholm, Sweden; E-Mail: anders.nordstrom@ki.se (A.N.); 4 Laboratory of Growth Regulators, Palacký University and Institute of Experimental Botany ASCR, Šlechtitelů 11, 783 71 Olomouc, Czech Republic; E-Mail: ondrej.novak@upol.cz (O.N.)

**Keywords:** cytokinin, deuterium labelling, biosynthetic rate, UPLC, MS

## Abstract

An improved method for determining the relative biosynthetic rate of isoprenoid cytokinins has been developed. A set of 11 relevant isoprenoid cytokinins, including zeatin isomers, was separated by ultra performance liquid chromatography in less than 6 min. The iP-type cytokinins were observed to give rise to a previously-unknown fragment at *m/z* 69; we suggest that the diagnostic (204-69) transition can be used to monitor the biosynthetic rate of isopentenyladenine. Furthermore, we found that by treating the cytokinin nucleotides with alkaline phosphatase prior to analysis, the sensitivity of the detection process could be increased. In addition, derivatization (propionylation) improved the ESI-MS response by increasing the analytes' hydrophobicity. Indeed, the ESI-MS response of propionylated isopentenyladenosine was about 34% higher than that of its underivatized counterpart. Moreover, the response of the derivatized zeatin ribosides was about 75% higher than that of underivatized zeatin ribosides. Finally, we created a web-based calculator (IZOTOP) that facilitates MS/MS data processing and offer it freely to the research community.

## 1. Introduction

The cytokinins are a group of plant hormones that play a central role in the regulation of cell division and differentiation. They control processes as diverse as apical dominance, root formation, leaf senescence, stomatal behavior, and chloroplast development [1]. Although their biological significance has been known for decades, the basic molecular mechanisms of cytokinin action have only recently been elucidated [2]. Structurally, cytokinins are adenine derivatives containing either an isoprenoid or an aromatic chain at the N6 position of the adenine ring. They mainly occur as free bases, nucleosides, nucleotides, and as a number of sugar conjugates substituted at the N7 and N9 positions of the purine ring (*N*-glucosides) or at the hydroxylated side chain (*O*-glucosides). However, only the free bases are thought to have significant biological activity. Accordingly, the interaction between the cytokinin ligand and its receptor is strongly influenced by the character of the side-chain. Nevertheless, cytokinin-receptor assays have shown that at least some receptors also respond to nucleosides and nucleotides [3].

The first dedicated step in cytokinin biosynthesis - *N*-prenylation of adenosine 5’-phosphates (AMP, ADP or ATP) with dimethylallyl diphosphate or hydroxymethylbutenyl diphosphate as side-chain donor - is catalyzed by the appropriate isopentenyltransferase (IPT; EC 2.5.1.27). The substrate specificity of IPTs varies depending on their origin and the species from which they are derived. In *Arabidopsis thaliana*, IPTs predominantly use ADP or ATP rather than AMP as prenyl acceptors, forming isopentenyladenosine 5’-diphosphate and 5’-triphosphate [4,5]. The isopentenyl side-chain can subsequently be *trans*-hydroxylated by cytochrome P450 monooxygenases to form *trans*-zeatin-type nucleotides [6]. In addition, tRNA IPTs, another group of cytokinin-generating enzymes, catalyze the prenylation of specific tRNAs and are responsible for the production of cis-zeatin-type cytokinins [7].

Plant tissue extracts are complex multi-component mixtures that contain cytokinins in minute quantities (fmol to pmol per gram of fresh weight) along with other compounds with similar structures and/or physico-chemical properties. Therefore, mapping the cytokinin composition of plants requires the use of sensitive and highly selective analytical methods. Hyphenated techniques such as ultra performance liquid chromatography-tandem mass spectrometry (UPLC-MS/MS) and capillary electrophoresis-tandem mass spectrometry (CE-MS/MS) have revolutionized trace analysis of cytokinins [8]. These methods combine a high-resolution separation system with a powerful detection and characterization technique. A high resolving power is necessary to separate cytokinin isomers, which differ significantly in their biological activity; mass spectrometric detection is essential because the sample matrix contains potentially interfering compounds at much higher concentrations than the target analytes.

The most common method to assess biosynthetic activity in biological systems involves the use of labeled precursors. Previously, both radioactive and stable-isotope precursors have been applied to elucidate cytokinin biosynthesis in various plant species [9,10]. Because the downstream analyses, scintillation and mass spectrometry, are very sensitive, labeled precursors can be added to the biological material at very low concentrations. This represents a significant advantage over classic feeding experiments, which use labeled precursors in concentrations high enough to trigger the activation of catabolic or interconversion enzymes and thus alter the metabolism of the studied compounds. Åstot and co-workers used an alternative method –*in vivo* deuterium labeling [11]. By growing plants on liquid cultivation media containing 30% D_2_O, deuterium was incorporated into general metabolic pathways and newly synthesized molecules thus labeled without altering their steady state levels [12]. The cytokinins were then isolated and the extent of their labeling was analyzed by liquid chromatography/frit-fast atom bombardment mass spectrometry. To increase the hydrophobicity of the target molecules, cytokinins were derivatized by propionylation prior to the final analysis [13]. A modified version of this approach, in which fast-atom bombardment was replaced by electrospray ionization, was subsequently used to study the crosstalk between auxins and cytokinins [14]. In the work described in this paper, we have focused on isoprenoid cytokinin bases, nucleosides and nucleotides (Table 1). We improved the method described above by using ultra performance liquid chromatography to separate 11 relevant cytokinins, including zeatin isomers.

**Table 1:** Structures, common names and abbreviations of the compounds used in this study. 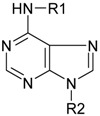

R_1_	R_2_	Common name	Abbreviation
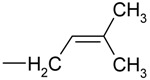	H	isopentenyladenine	iP
	R	isopentenyladenosine	iPR
	RMP	isopentenyladenosine-5´-monophosphate	iPMP
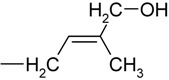	H	*trans*-zeatin	*t*Z
	R	*trans*-zeatin riboside **	*t*ZR
	RMP	*trans*-zeatin riboside-5´-monophosphate	*t*ZMP
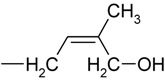	H	*cis*-zeatin**	*c*Z**
	R	*cis*-zeatin riboside	*c*ZR
	RMP	*cis*-zeatin riboside-5´-monophosphate	*c*ZMP
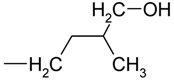	H	dihydrozeatin	DHZ
	R	dihydrozeatin riboside	DHZR

H: hydrogen; R: β-D-ribose; RMP: β-D-ribose-5´-monophosphate.

In addition, by treating the cytokinin nucleotides with alkaline phosphatase followed by immunoaffinity purification of the dephosphorylated ribosides, we increased the sensitivity of the mass spectrometric detection. This method was used to measure the rate of synthesis of selected isoprenoid cytokinins isolated from the cytokinin‑overproducing Arabidopsis line *PGA22*, and the results so obtained were compared to those obtained using an alternative method based on the analysis of underivatized cytokinins. Finally, we created a web-based calculator to process mass spectrometric data and convert them to tracer:tracee ratios (t/t), as described by Åstot *et al.* [11].

## 2. Results and Discussion

### 2.1. Implementation of ultra performance liquid chromatography expedites cytokinin analysis

As analytical methods evolve, it becomes necessary to perform increasing numbers of separations. To make it easier to cope with this growing need, we sought to improve on the current HPLC method for the separation of propionylated cytokinins [11]. Generally, there are three main approaches to reducing the time required for a given LC separation: (i) the use of monolith columns, (ii) liquid chromatography at high temperatures and (iii) liquid chromatography at ultra-high pressures using columns packed with sub-2-micron particles (UPLC) [15]. We have previously used UPLC with a 50 mm column (BEH C18, 50 × 2.1 mm; 1.7 μm particle size; Waters) and 15% ammonium formate/methanol as the mobile phase buffers to separate 21 underivatized cytokinins [17]. However, isomer co-elution made it impossible to separate propionylated cytokinins under these chromatographic conditions. The various isomers of the zeatin-type cytokinins have identical molecular masses and give rise to identical basic fragments under MS/MS conditions, highlighting the need for efficient methods for their chromatographic separation.

**Figure 1:**
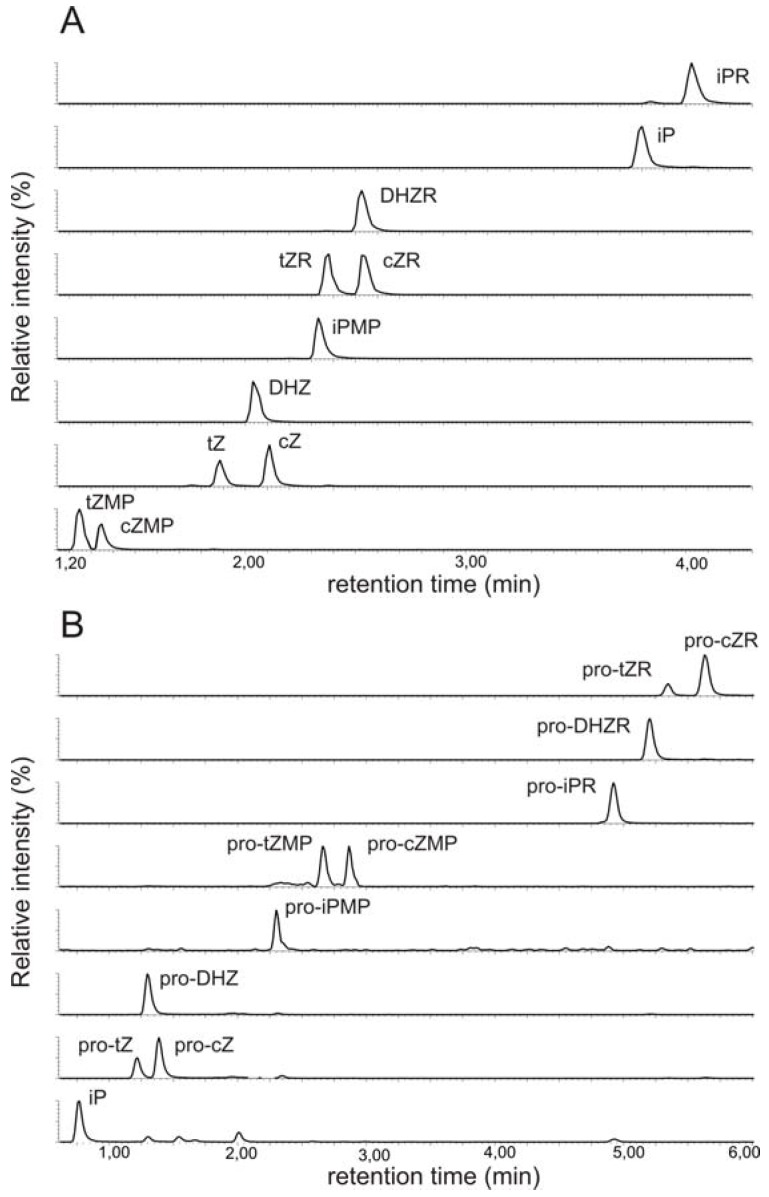
Separation of a mixture of cytokinin standards by ultra-performance liquid chromatography (UPLC). The figure shows reconstituted mass chromatograms of 11 underivatized (A) and 11 propionylated (B) cytokinins; each trace represents one MRM transition. The mixture contained 1 pmol of each metabolite.

On the basis of this experience, we sought to develop a UPLC method using the same 50 mm column for the separation of propionylated cytokinins. A gradient elution program was designed using the Acquity UPLC columns calculator (Masslynx v.4.1, Waters) and slightly adjusted to achieve a baseline separation of 11 isoprenoid cytokinins, including free bases, nucleosides and nucleotides. The HPLC method previously used for such separations employed a 150 mm column and took 56 min; the new UPLC method uses a 50 mm column and has a chromatographic separation time of less than 6 min. The gradient elution buffers used in this method (water and acetonitrile) typically contain 3% of added formic acid [16], which causes ion-source pollution. This decreases the sensitivity of the mass spectrometer and necessitates daily cleaning of the ion-source. By reducing the formic acid content of the mobile phase to 0.7%, we were able to significantly reduce the contamination of the mass spectrometer ion-source without affecting the quality of the chromatographic separation. Decreasing the formic acid content of the mobile phases to less than 0.7% or raising their pH resulted in the loss of chromatographic resolution. Although the selectivity of this chromatographic system differs from that of the system developed for the separation of underivatized cytokinins, both approaches can be used to separate 11 cytokinins in less than 6 min (Figure 1).

### 2.2. Mass spectrometric detection

**Figure 2:**
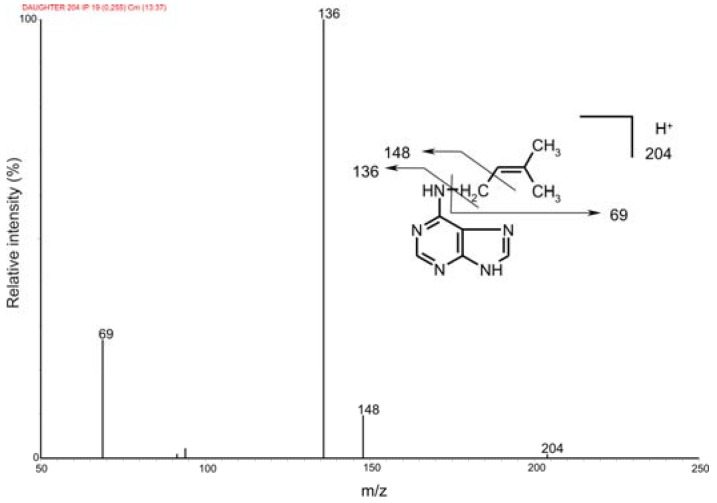
Collision mass spectrum of isopentenyladenine and its fragmentation pattern.

We began by recording positive electrospray-ionization mass spectra for all 11 propionylated cytokinins. The background-subtracted ESI+ spectra of all of the studied compounds contained base peaks corresponding to the quasi-molecular ions [M+H]+ and no adduct formation was observed under the acidic conditions (data not shown). Moreover, the fragmentation patterns were consistent with previously acquired FAB MS spectra [13]. However, we observed a previously neglected sidechain fragment at *m/z* 69 in the spectra of various iP-type cytokinins, including the free bases, ribotides, ribosides and glucosides (Figure 2).

To confirm the identity of this fragment we performed an exact mass determination of the fragments of isopentenyladenine (iP) and its deuterium-labeled counterpart (D_6_-iP) by quadrupole-time-of-flight mass spectrometry. We reasoned that if unlabelled iP gives a side-chain fragmnent at *m/z* 69, D_6_-iP should give rise to an analogous fragment at *m/z* 75 due to the incorporation of six deuterons into the terminal methyl groups of the isopentenyl moiety. The results obtained are summarized in Table 2.

**Table 2:** The identification of an isopentenyladenine side-chain fragment by exact mass determination using a Q-TOF mass spectrometer. Experimental values are compared to those calculated from the fragments' chemical formulae. The difference between the two values (in ppm) was calculated as a measure of fidelity.

Features	iP	D_6_-iP
Selected fragment formula	C_5_H_9_^+^	C_5_H_3_D_6_^+^
Experimental *m/z*	69.0703	75.1081
Calculated *m/z*	69.0704	75.1081
Difference (ppm)	-1.4	0.0

The diagnostic transition 204-69 could potentially be used to monitor the labeling of isopentenyladenine with deuterium. It is better suited for this purpose than is the 204-136 transition, whose magnitude reflects the extent of deuterium incorporation into the adenine moiety rather than the labeling of the sidechain; while labeling of the adenine moiety may be of interest in other contexts, it is not relevant in studies of cytokinin biosynthesis. The diagnostic quasi-molecular ion – fragment ion transition at *m/z* 69 was observed in the mass spectra of other iP type cytokinins and so may be of use as a general indicator of deuterium labeling in these species. Because the focus of this study was on the primary products of cytokinin biosynthesis, *i.e*. cytokinin nucleotides and their ribosides, we did not investigate the extent of deuterium incorporation into the free bases.

The *in vivo* deuterium labeling method exploits the ability of intact plants to take up the tracer, which easily penetrates all cell compartments and enters the general metabolic pathways, giving rise to labeled precursors. Åstot and co-workers showed that the extent to which deuterium is incorporated into the main structural components of the cytokinins depends on the relative biosynthetic rates of the different pathways that produce them (note that “biosynthetic rate” here and throughout this paper refers to the measured degree of labeling of the compound rather than the exact rate of its biosynthesis) [11]. Labeling was observed in the propionyl ribose, the cytokinin base, and the isoprenoid side-chain. Since the first step in cytokinin biosynthesis is the prenylation of ADP and/or ATP leading to the formation of isopentenyladenosine-5'-diphosphate and/or isopentenyladenosine-5'-triphosphate [4,5], we selected the quasi-molecular ion of pro-iPR at *m/z* 504 as the precursor ion and the free base fragment at *m/z* 204 as the product ion for multiple reaction monitoring of isotopomer I0 (the isotopomer with lowest *m/z*), to exclude the signal of the ribose moiety. The isopentenyl moiety can be trans-hydroxylated by cytochrome P450 monooxygenases to form trans-zeatin-type nucleotides [6]. The rate of biosynthesis of the zeatin-type cytokinins was monitored using the analogous *m/z* 576-276 diagnostic transition for the I0 isotopomer. This transition corresponds to the dissociation of pro-zeatin riboside to its aglycone. An overview of the diagnostic transitions examined in this work is given in Table 3.

**Table 3:** Diagnostic transitions used to measure the rate of synthesis of selected cytokinins.

Derivatized cytokinins			Underivatized cytokinins		
Cytokinin	Isotopomer	Diagnostic transition	Cytokinin	Isotopomer	Diagnostic transition
pro-iPR	*I_0_*	504-204	iPR	*I_0_*	336-204
	*I_1_*	505-205		*I_1_*	337-205
	*I_2_*	506-206		*I_2_*	338-206
	*I_3_*	507-207		*I_3_*	339-207
pro-ZR	*I_0_*	576-276	ZR	*I_0_*	352-220
	*I_1_*	577-277		*I_1_*	353-221
	*I_2_*	578-278		*I_2_*	354-222
	*I_3_*	579-279		*I_3_*	355-223

### 2.3. Enzymatic treatment of cytokinin nucleotides improves the sensitivity of mass spectrometric detection

**Figure 3:**
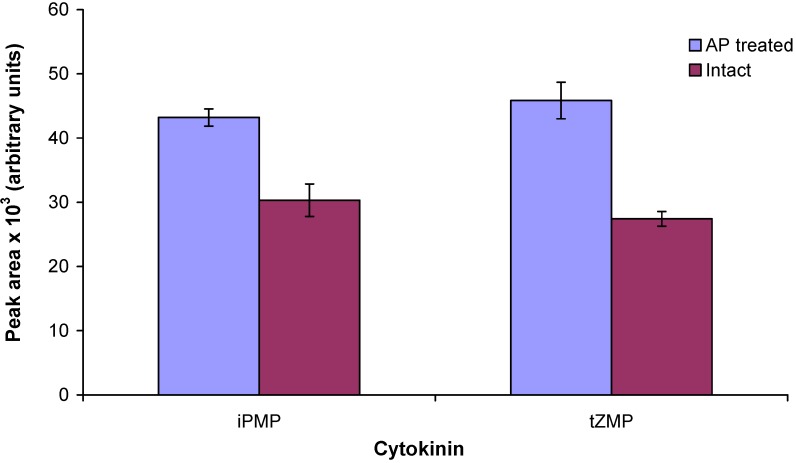
Cytokinin nucleotides analyzed by UPLC-MS/MS as intact monophosphates and after AP treatment. 0.5 pmol of each cytokinin was injected onto the chromatographic column and detected by MS using multiple reaction monitoring mode. The intensities of the mass spectrometric signals corresponding to the first isotopomer (I0) are shown.

Generally, cytokinin nucleotides can be analyzed by LC-MS either as intact monophosphates [16,17] or as the corresponding ribosides, prepared by treatment with alkaline phosphatase (AP) [18]. The ribosides can be further purified by immunoaffinity chromatography using a broad-spectrum monoclonal anti-cytokinin antibody that recognizes free bases, 9-ribosides and 9-glucosides but not the nucleotides (L. Spichal, unpublished data). To determine which approach gives the best sensitivity, we prepared two sets of cytokinin standards (5 nmol of tZMP and iPMP). The first set of samples (five replicates) was treated with alkaline phosphatase, the reaction products were purified by immunoaffinity chromatography (IAC) and the eluate was evaporated to the dryness. The second set of samples was directly evaporated to dryness and both sets were derivatized by propionylation [13]. All samples were re-dissolved in the initial mobile phase and 0.5 pmol of each analyte was injected onto the chromatographic column. The samples were analyzed by the UPLC-MS/MS method described above and the signals corresponding to the first isotopomer (I0) in each case were compared (Figure 3). The signals of iPMP and tZMP treated with alkaline phosphatase were about 42% and 67% higher than those of the non-treated nucleotides, respectively, which is in good agreement with our previously published data on the quantitative analysis of cytokinins [16]. Generally, the ESI response is higher for more hydrophobic molecules because they have a greater affinity for the droplet surface [19]. Because cytokinin ribosides are more hydrophobic than cytokinin monophosphates, their detection limit is almost 5-fold lower [16]. Moreover, propionylation of cytokinin ribosides gave slightly higher yields (data not shown). On the other hand, the recovery of IAC ranges between 30 and 70% [17]. Altogether, the data indicate that the use of enzymatic treatment and purification by IAC significantly improves the ESI-MS signal. Additionally, when AP treatment is omitted, only the monophosphates are detected and analyzed. However, experiments using recombinant IPTs suggest that the primary products of CK biosynthesis are isopentenyladenosine 5’-diphosphate and 5’-triphosphate [4,5]. AP will cleave all of the various forms of the nucleotides (*i.e.* mono-, di-, and tri-phosphates), making it possible to analyze all of them at once; while this approach is obviously less informative than an analysis of the individual nucleotides, it is very convenient and efficient. We recently published a HPLC-MS method for the determination of underivatized intact cytokinin nucleotides in human leukemia cells [20]. However, the sensitivity of this method is restricted by the poor ionization efficiency of cytokinin di- and tri-phosphates. Moreover, an efficient procedure for the purification of cytokinin nucleotides isolated from plant tissue remains to be developed; current purification methods offer recoveries ranging from 34% to 65% [16,17]. It is evident that despite ongoing progress in method development, an optimal technique for the analysis of individual cytokinin nucleotides isolated from plant tissue is not yet available.

### 2.4. Application of the new method

**Figure 4:**
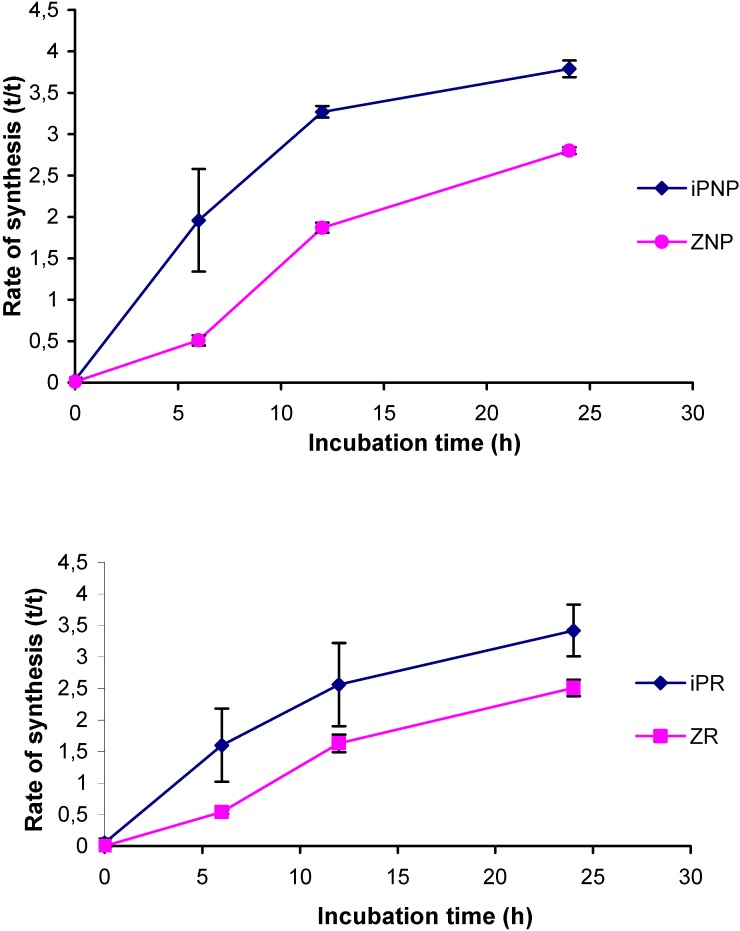
The biosynthetic rate (tracer:tracee ratio) of iP-type nucleotides (IPNP), tZ-type nucleotides (tZNP), iPR and tZR isolated from Arabidopsis line *pga22* plotted against the incubation time. Data were acquired in MRM mode and include corrections to compensate for the natural isotope distribution.

The newly-developed UPLC-MS/MS method was employed for the analysis of the relative rate of biosynthesis of cytokinins in the cytokinin-overproducing Arabidopsis line *pga22*, which carries the AtIPT8 gene under the control of the 17-β-estradiol-inducible promoter/enhancer [20]. It has previously been reported that over the course of a 24-hour induction period, iPMP and iPR levels in this mutant increased more than 19- and 38-fold, respectively, but only minor increases in the levels of zeatin-type monophosphates and ribosides were observed [20]. Three-week-old Arabidopsis seedlings were incubated in liquid growth medium enriched with 30% deuterium oxide with 0 (control) or 5 μM 17-β-estradiol for 24 hours, after which the nucleotides were extracted, subjected to AP treatment and purification by IAC, and analyzed. Cytokinins including iPNP, tZNP, iPR and tZR, showed significant deuterium enrichments, *i.e.* increased ratios of labeled to unlabeled cytokinin after correcting for the natural isotope distribution (tracer/tracee ratio; Figure 4). 

As expected, induction of AtIPT8 expression triggered an increase in the biosynthetic rate. The iP-type nucleotides (iPNP) were more extensively labeled than were the tZ-type nucleotides (tZNP) throughout the experiment. This is consistent with iPNPs being the primary products of IPT action and tZNP being synthesized from them by trans-hydroxylation. In addition, both ribosides were less extensively labeled than their phosphorylated counterparts, confirming that *in vivo* dephosphorylation occurs at a relatively late stage in cytokinin biosynthesis. Interestingly, the absolute t/t-ratios were similar to those obtained in a bacterial IPT overexpression system [12]. The tracer:tracee ratio of the *cis-*Z-type cytokinins remained at the basal level throughout the experiment (data not shown).

### 2.5. Comparison to existing methods

**Figure 5:**
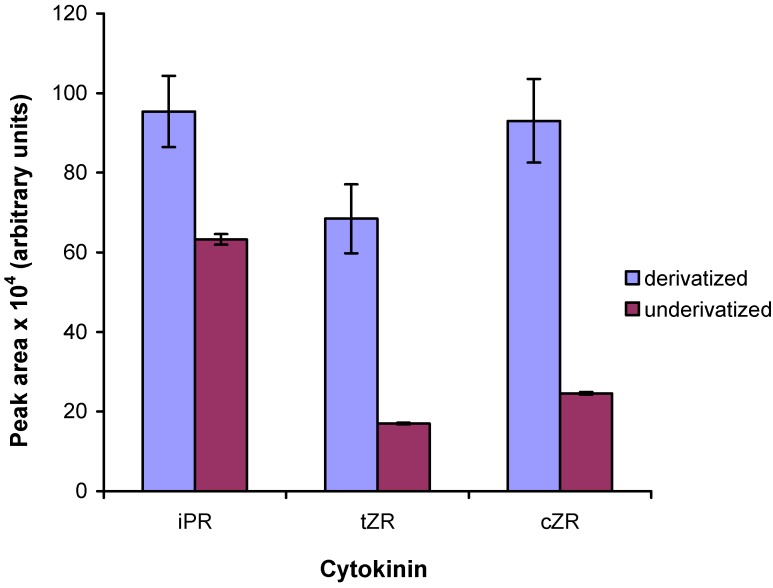
Comparison of ESI-MS response. 10 pmol of derivatized and underivatized cytokinin standards were analyzed using UPLC-MS/MS. Peak areas (arbitrary units) of the first isotopomer are compared. Error bars indicate the standard deviation (n=4).

Finally, we compared the newly-developed UPLC-MS/MS method to that developed by Dobrev and co-workers, [21] which was designed for the analysis of underivatized cytokinins. The underivatized cytokinins were resolved on a BEH C18 chromatographic column (50 × 2.1 mm, particle size 1.7 μm; Waters) using 15 mM ammonium formate (pH 4) and methanol as mobile phase buffers [17]. The diagnostic transitions utilized for mass spectrometric detection of derivatized and underivatized cytokinins are summarized in Table 3. A quasi-molecular ion of the protonated riboside was selected as the precursor ion, and the most intense fragment (a free base) was selected as the product ion for multiple reaction monitoring of isotopomer I0. This diagnostic transition allowed us to exclude signals arising from the ribose moiety. The response of underivatized iPR was about 34% lower than that of its propionylated counterpart. The response of the underivatized zeatins was about 75% lower than those of propionylated zeatins (Figure 5). The difference between the values obtained for both cytokinin types reflects the fact that the different analyte molecules incorporate different numbers of propionyl groups during derivatization: whereas iPR carries propionyl groups on the ribose moiety only, zeatins carried an additional one on the hydroxylated side-chain. Derivatization increased the ESI response of the analytes by augmenting their hydrophobicity and also increased their molecular mass. This increase in mass shifted the signals arising from the derivatized molecules into an *m/z* region with relatively little chemical noise, resulting in a slightly higher MS-response. The sensitivity of MS detection is very important when designing specific biological experiments. Previously, at least 1 g of fresh plant tissue was required for measurements of the cytokinin biosynthetic rate [22], which is problematic when valuable information on tissue and organ specific cytokinin biosynthesis is desired. For the sake of comparison, the concentrations of the auxins (another class of phytohormones) in plant tissues are more than ten times higher than those of cytokinins and consequently, only a few miligrams of fresh plant material is necessary for accurate measurements of the biosynthetic rate of auxins, making experimental resolution of auxin concentrations at the cellular level possible [23]. Although, the proposed UPLC-MS/MS method for cytokinins cannot be used for analysis at the cellular level, the organ level would be attainable if one were to use other, larger plants than Arabidopsis. Derivatization thus increases the sensitivity of mass spectrometric detection to the point that much less plant tissue is required for analysis.

Both approaches, with or without derivatization, can be used to measure the relative biosynthetic rate of cytokinins. In both cases, analysis of peaks corresponding to the loss of the ribose moiety should yield similar results. To confirm this assumption we measured the relative biosynthetic rates of iPNP and iPR in the *pga22* line by both methods. Cytokinins were isolated by harvesting Arabidopsis seedlings 6 hours after chemical induction of AtIPT8, fractionating them to separate the ribosides from the nucleotides, treating the latter with AP, and finally purifying them by IAC. The results are summarized in Table 4.

**Table 4:** The relative biosynthetic rate (t/t) of iP-type nucleotides (iPNP) and isopentenyladenosine (iPR) isolated from Arabisopsis seedlings 6h after chemical induction of *AtIPT8*.

Cytokinin	Derivatized		Underivatized	
	Rate of synthesis (t/t)	SD	Rate of synthesis (t/t)	SD
iPNP	1.6843	0.2099	1.7961	0.1750
iPR	1.2965	0.1334	1.4538	0.1525

As expected, no significant differences were observed in the relative biosynthetic rates of iP-type cytokinins. Both UPLC-MS/MS methods are rapid, achieving complete separation in less than 6 min. Sample preparation remains the most time consuming part of the entire method; the derivatization in particular requires at least 10 hours (encompassing derivatization, incubation, and concentration *in vacuo*). Nonetheless, this step is worthwhile because it greatly increases the sensitivity of the mass spectrometric detection. Since all of the relevant cytokinin metabolites are well resolved by UPLC, it is possible to analyze the whole set at once if necessary. The method desribed in this paper could potentially be used to analyze deuterium enrichment in all of the various kinds of cytokinins, including nucleotides, ribosides, free bases and glucosides, provided that one could obtain enough material. In this work, we decided to study the rather small plant *Arabidopsis thaliana,* which is a very popular model organism in plant sciences. However, due to the low mass of the plant seedlings and the relatively low levels of physiologically active CK bases, we chose to focus exclusively on nucleotides and ribosides in this instance.

We have also created a web-based calculator (IZOTOP), which helps the user to convert signals obtained by MS/MS measurements into relative biosynthetic rate (t/t ratio) values. The procedure used in performing these calculations was adapted from the work of Åstot *et al*. [11]. This calculator is freely available (www.biochemie.upol.cz/software/izotop).

## 3. Experimental

### 3.1. Chemicals

Authentic cytokinin standards were purchased from Olchemim (Olomouc, Czech Republic), formic acid from Merck (Darmstadt, Germany), and DEAE-Sephadex A-25 from GE Healthcare (Uppsala, Sweden). Deionised (Milli-Q) water was obtained from a Simplicity 185 water system (Millipore, Bedford, MA, USA). All other chemicals were of analytical grade or higher purity and were purchased from Sigma-Aldrich Chemie (Steinheim, Germany). 

### 3.2. Biological material

*Arabidopsis thaliana* line *pga22* carrying the *AtIPT8* gene under the control of the 17-β-estradiol-inducible promoter/enhancer [18] was grown in 250-mL Erlenmeyer flasks containing 50 mL of full Murashige and Skoog (MS) basal growth medium, 3% sucrose, pH 5.6 (25 seeds per flask). The flasks were agitated and maintained at 23 °C under long day conditions with 18 h light and 6 h darkness. After three weeks, the plants were transferred to half-strength MS medium containing 1.5% sucrose, 30% deuterium oxide and 0 or 5 μΜ 17- β -estradiol to induce cytokinin biosynthesis. 

### 3.3. Sample preparation

Samples were extracted and purified essentially as outlined by Novák *et al.* [18]. Briefly, frozen plant material (500 mg fresh weight) was homogenized using an MM 301 vibration mill (Retsch, Haan, Germany) at a frequency of 30 Hz for 2 min and extracted overnight in methanol–chloroform–formic acid–water (12:5:1:2, v/v/v/v). The extract was first passed through a cation (SCX-cartridge) and then an anion [DEAE-Sephadex combined with an SPE(C18)-cartridge] exchanger to yield fraction 1, which contained the cytokinin free bases, ribosides, and glucosides, and fraction 2, which contained the riboside-5'-phosphates. Fraction 2 was first treated with alkaline phophatase (4U/sample, 37 °C, 60 min) and both fractions were further purified by immunoaffinity chromatography based on generic monoclonal anticytokinin-antibodies. For propionylation, the samples were dissolved in 10 μL of dimethylformamide. 6 μL of N-methylimidazole and 2 μL of propionic anhydride were added and the reaction mixtures were heated at 37 °C for 60 min, after which they were evaporated under reduced pressure.

### 3.4. HPLC-Q-TOF MS identification

A hybrid Q-TOF micro^TM^ mass spectrometer (Waters MS Technologies) was used for the high-resolution identification and confirmation of the side-chain fragments at *m/z* 69 and *m/z* 75. Electrospray ionization in the positive ion mode was used with the following parameters: source block/desolvation temperature, 100 °C/350 °C; capillary/cone voltage, 2500/25 V; and spray/cone gas flow (N_2_), 500/50 L/h. In the product ion scan of the selected precursors (*m/z* 204 and *m/z* 210), data were acquired in the mass range *m/z* 50–250, with a cycle time of 33 ms, a scan time of 2.0 s, and collision energy of 40 eV, 50 eV and 60 eV. For the exact mass determination experiments, a lock spray was used for external calibration with a mixture of 0.1 M NaOH/10% formic acid (v/v) and acetonitrile (1:1:8, v/v/v) as a reference. Accurate masses were calculated and used for the determination of the elementary composition of the analytes with fidelity of 5 ppm.

### 3.5. UPLC-MS/MS

An Acquity UPLC™ System (Waters, Milford, MA, USA) coupled to a Xevo triple-stage quadrupole mass spectrometer (Waters MS Technologies, Manchester, UK) was utilized for the cytokinin measurements. The system was controlled by Masslynx software (version 4.0, Waters, Manchester, UK).

Derivatized cytokinins were separated on a chromatographic column (BEH C18, 50 × 2.1 mm, particle size 1.7 μm; Waters) using 0.7% HCOOH in water and 0.7% HCOOH in acetonitrile as solvents A and B, respectively. At a flow-rate of 0.5 mL min^-1^, the following elution profile was used: an isocratic step at 15% B for 0.5 min preceded a linear gradient from 15 to 45% B for 5.5 min. At the end of the gradient there was a washing step at 100% B (for 2 min) and then the column was equilibrated to the initial conditions for 1.9 min. The column was thermostated at 40 °C. Tandem mass spectra of all cytokinins examined in this study were acquired by continuous infusion of 10^-3^ mol L^-1^ solution in solvent A/solvent B (1:1, v/v) at a flow-rate of 15 μL min^-1^.The capillary voltage, cone voltage, collision cell energy, and ion source temperature were optimized for each individual compound. The mass spectrometer settings were as follows: a capillary voltage of 3 kV, a cone voltage of 33 V, a source temperature of 120 °C, a desolvation temperature of 550 °C, a cone gas flow of 70 L h^-1, a desolvation gas flow of 600 L h-1^, collision cell energy of 19 eV, and a collision gas flow of 0.2 mL min^-1^.

Underivatized cytokinins were separated on chromatographic column (BEH C18, 50 × 2.1 mm, particle size 1.7 μm; Waters) using 15 mM HCOOH (pH 4.0, adjusted by NH_4_OH) and methanol as solvent A and B, respectively. At a flow-rate of 0.5 mL min^-1^, the following elution profile was used: an isocratic step at 10% B for 0.5 min preceded a linear gradient from 12 to 45% B for 3.5 min. At the end of the gradient there was a washing step at 100% B (for 0.3 min) and then the column was equilibrated to the initial conditions for 1.2 min. The column was thermostated at 40 °C. The capillary voltage, cone voltage, collision cell energy, and ion source temperature were optimized for each individual compound. The mass spectrometer settings were as follows: a capillary voltage of 3 kV, a cone voltage of 30 V, a source temperature of 120 °C, a desolvation temperature of 575 °C, a cone gas flow of 70 L h^-1, a desolvation gas flow of 600 L h-1^, collision cell energy of 19 eV, and a collision gas flow of 0.2 mL min^-1^.

## 4. Conclusions

We have developed a UPLC-MS/MS method for determining the relative rates of biosynthesis of cytokinin-type phytohormones. The method is based on a rapid and efficient separation of derivatized analytes by ultra-performance liquid chromatography combined with sensitive and selective mass spectrometric detection. We show that the biosynthetic rate of isopentenyladenine can be measured using a previously neglected diagnostic 204-69 transition. Cytokinin nucleotides are analyzed after treatment with alkaline phosphatase, which enhances the sensitivity of the detection process. The new method proved to be more sensitive than one based on the analysis of underivatized cytokinins, although the relative synthetic rate values measured by the two both methods were similar. Finally, we created a web-based calculator (IZOTOP) that facilitates MS/MS data processing and have made it freely available to the research community. 
